# Efficacy and Safety of IgPro20, a Subcutaneous Immunoglobulin, in Japanese Patients with Primary Immunodeficiency Diseases

**DOI:** 10.1007/s10875-013-9985-z

**Published:** 2014-02-07

**Authors:** Hirokazu Kanegane, Kohsuke Imai, Masafumi Yamada, Hidetoshi Takada, Tadashi Ariga, Martin Bexon, Mikhail Rojavin, Wilson Hu, Midori Kobayashi, John-Philip Lawo, Shigeaki Nonoyama, Toshiro Hara, Toshio Miyawaki

**Affiliations:** 1 Department of Pediatrics, Graduate School of Medicine and Pharmaceutical Sciences, University of Toyama, Toyama, Japan; 2 Department of Community Pediatrics, Perinatal and Maternal Medicine, Tokyo Medical and Dental University, Tokyo, Japan; 3 Department of Pediatrics, Hokkaido University Graduate School of Medicine, Sapporo, Japan; 4 Department of Pediatrics, Graduate School of Medical Sciences, Kyushu University, Fukuoka, Japan; 5 CSL Behring AG, Berne, Switzerland; 6 CSL Behring LLC, King of Prussia, PA USA; 7 CSL Limited, Research and Development, Melbourne, Victoria Australia; 8 CSL Behring KK, Tokyo, Japan; 9 CSL Behring GmbH, Marburg, Germany; 10 Department of Pediatrics, National Defense Medical College, Saitama, Japan; 11 Toyama City Hospital, Toyama, Japan

**Keywords:** Primary immunodeficiency, PID, primary antibody deficiency, SCIG, Hizentra®, IgPro20, Japan

## Abstract

**Purpose:**

Intravenous (IVIG) and subcutaneous (SCIG) immunoglobulin infusions are widely used for the treatment of patients with primary immunodeficiency (PID) worldwide. This prospective, multicenter, open-label, single-arm Phase III study evaluated the efficacy, tolerability, and safety of IgPro20 (Hizentra®; L-proline–stabilized 20 % human SCIG) in adult and pediatric Japanese patients with PID.

**Methods:**

Patients received three IVIG infusions at 3–4-week intervals followed by a dose-equivalent switch to weekly SCIG infusions. A 12-week wash-in/wash-out period was followed by a 12-week SCIG efficacy period. The primary efficacy endpoint was the comparison of total serum IgG trough levels during the IVIG and SCIG efficacy periods by calculating the geometric mean ratio (GMR).

**Results:**

The GMR of IgG trough levels on SCIG versus IVIG was 1.09 (2-sided 90 % confidence interval: 1.06–1.13). No serious bacterial infections were reported. Eleven patients (52.4 %) had infectious episodes with an overall rate of 2.98 infections/patient/year; 7 patients (33.3 %) missed school/work/daycare due to infection (3.48 days/patient/year). Sixteen patients (76.2 %) were treated with antibiotics for an adverse event (AE; 47.6 %) or prophylaxis (23.8 %), resulting in 167.42 days/patient/year of antibiotic use. During SCIG treatment, 24 patients (96.0 %) had 269 AEs (0.461 AEs per/infusion) including local reactions as the most common AE (20 patients, 80.0 %). Local tolerability of IgPro20 was assessed as “very good” or “good” after 85.4 % of SCIG infusions. One patient (4.0 %) experienced a serious AE of moderate severity (bacterial infection) that was considered unrelated to study medication.

**Conclusion:**

IgPro20 was effective and well tolerated in Japanese patients with PID.

## Introduction

Primary immunodeficiency (PID) includes a range of genetic disorders that are characterized by an intrinsic defect in the immune system (B- and T-cell defects, phagocytic disorders, and complement deficiencies) [1]. PID results in the patient’s predisposition to recurrent multiple infections despite intensive treatment with antibiotics [2, 3].

Prevalence of PID in Japan was determined in a recent nationwide survey (*N* = 1,240) to be 2.3 PID patients per 100,000 inhabitants, with minimal variations across the country regions [4]. These results are comparable with the prevalence of PID in Taiwan (0.77–2.17) and Singapore (2.7), but are lower than those in European countries (e.g., 4.4 in France), the Middle East (11.98), and the US (83.3) [4, 5].

Most patients with PID have a primary antibody deficiency (PAD) and require immunoglobulin G (IgG) replacement therapy with regular administration of IgG to prevent infection and maintain quality of life [3]. Intravenous immunoglobulin (IVIG) infusions every 3–4 weeks are the current standard practice in Japan. However, the likelihood of developing severe and/or systemic adverse reactions and the difficulties in obtaining venous access, particularly in children, have prompted the development of alternative modes of IgG delivery. Consequently, IgG administration by the subcutaneous route (SCIG) has been developed and is now available in Europe and the United States [6, 7]. IVIG and SCIG infusions are the current treatment modalities of choice for patients with PAD worldwide [8, 9].

Patients and their doctors select SCIG for better flexibility and freedom from long visits to clinics for IVIG infusions. SCIG does not require venous access, which is particularly welcome in children. Additional medical benefits of SCIG compared with IVIG include lower rates of systemic adverse events (AEs) and more even serum IgG levels between the infusions [6, 7, 10]. The latter ensures better protection against infections over the full period between the infusions and helps to alleviate or completely avoid the known “wear-off” effects, such as decrease of an overall well-being and higher probability of infection development during the last pre-infusion week [6, 11].

IgPro20 (Hizentra®; L-proline–stabilized 20 % human SCIG) is the first and only 20 % SCIG preparation approved in the US (2010) and Europe (2011) for treatment of PID. It has been found effective and well tolerated in two Phase III studies in both adult and pediatric patients with PID [6, 7, 12–14].

This is the first prospective, multicenter, open-label, single-arm study of SCIG in the Japanese population. Here we report the results of efficacy, tolerability, and safety evaluation of SCIG therapy in Japanese patients with PID after a dose-equivalent switch from IVIG.

## Methods

### Patients, Inclusion and Exclusion Criteria

Male and female outpatients 75 years of age or younger with PID requiring IgG replacement therapy were eligible for the study. The PID diagnosis was based on the diagnostic criteria defined by the Pan-American Group for Immunodeficiency and the European Society for Immunodeficiencies [15]. Only patients who had received at least three doses of IVIG at regular 3- or 4-week intervals at a stable dose (variations of ±10 % were allowed) prior to enrollment were eligible. For inclusion in the SCIG period, the patient’s IgG trough levels between screening (or first mandatory IVIG infusion) and third mandatory IVIG infusion should have remained above or equal to 4 g/L, with at least one measurement above or equal to 5 g/L.

Patients who developed a serious bacterial infection (SBI) at the time of screening or during the mandatory IVIG treatment period were to be excluded from the study. Other exclusion criteria included lymphoid system malignancy, hyperprolinemia, known allergies or severe reactions to immunoglobulins or other blood products, hypoalbuminemia, protein-losing enteropathies, proteinuria (total urine protein concentration above 0.2 g/L), known hemophilia, or thrombocytopenia (platelet count below or equal to 50 × 10^9^/L). Oral or parenteral steroids were allowed at doses less than 0.15 mg of prednisone equivalent/kg/day. Women who were pregnant, breastfeeding, not using appropriate contraception, or planning a pregnancy during the course of the study were also excluded.

This study was conducted in accordance with the International Conference on Harmonisation Good Clinical Practice guidelines, and the Declaration of Helsinki (2008 version). The study protocol and all other study documents were approved by the relevant independent Ethics Committees. Signed written informed consent (written assent for patients 7 years of age or younger at the time of obtaining the assent) was obtained from the patients or their parents or legally acceptable representatives prior to any study-related activities. This study was registered at ClinicalTrials.gov (study identifier NCT01199705).

### Study Design

This prospective, multicenter, open-label, single-arm Phase III study was designed to evaluate the efficacy, tolerability, and safety of IgPro20 in Japanese patients with PID requiring IgG replacement therapy. The primary objective of the study was to assess whether total serum IgG trough levels achieved with preceding IVIG treatment could be sustained upon a dose-equivalent switch to IgPro20.

**Fig. 1:**
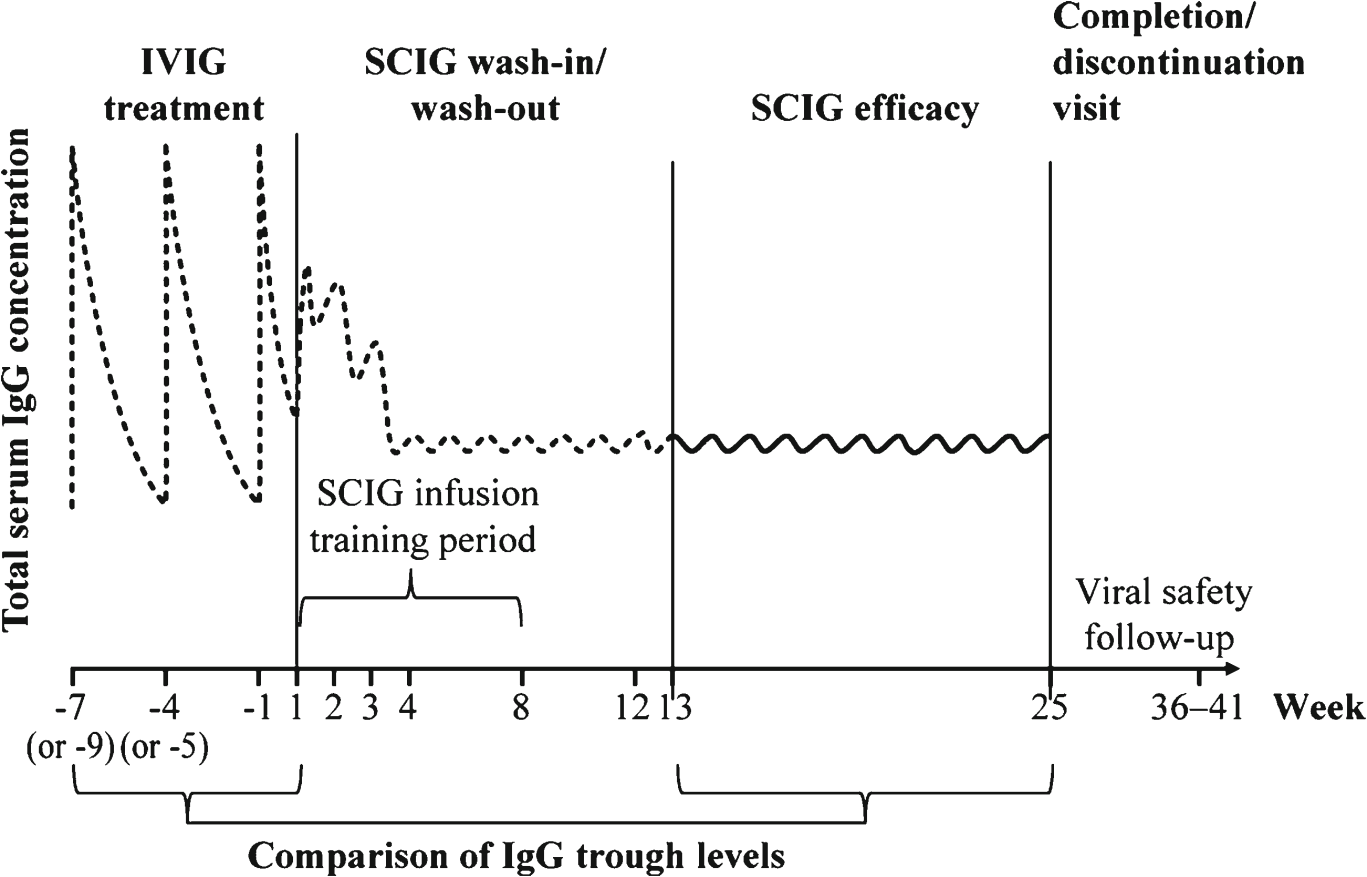
Study design. Patients received three mandatory IVIG infusions at 3- or 4-week intervals (times for 4-week intervals are shown in brackets), followed by 24 weekly SCIG infusions during the wash-in/wash-out period (Weeks 1–12) and SCIG efficacy period (Weeks 13–25). There were two follow-up visits: completion visit (Week 25) and viral safety follow-up visit 12–17 weeks after completion. The *horizontal curve* schematically represents the expected fluctuations of serum IgG levels before (*dashed curve*) and during the SCIG efficacy period (*solid curve*)

The study consisted of a screening period, a mandatory IVIG treatment period with three planned infusions at 3- or 4-week intervals at the same dose as the one administered prior to the study, a 12-week SCIG wash-in/wash-out period, and a 12-week SCIG efficacy period with weekly infusions followed by a completion or discontinuation visit (Fig. 1). For all patients, a viral safety follow-up visit was performed 12–17 weeks after the last SCIG infusion.

Weekly SCIG infusions were allowed to be done at home, except for the first 3 to 8 infusions conducted at the study site. These supervised SCIG infusions were provided to train the patient or their parent or legally acceptable representative who performed the rest of home-based SCIG infusions.

IgPro20 was infused at predefined injection sites on the upper arms, abdomen, thighs, and/or lateral hip recommended by the investigator. The maximum volume per injection site was 25 mL. The actual point(s) of injection could be changed, if needed, with each weekly administration. In case of injection-related local reactions, it was not recommended to use a certain injection site until the local reaction from the previous injection had completely resolved.

To provide a dose-equivalent switch to IgPro20, the initial weekly dose of IgPro20 during the wash-in/wash-out period was calculated as the previous IVIG dose divided by the IVIG dosing interval in weeks. If the IVIG dose had been adjusted during the mandatory IVIG treatment period, the average of the three actual doses was used for calculations. The initial IgPro20 dose could be adjusted, if necessary, based on the IgG trough levels measured during the wash-in/wash-out period, to achieve IgG trough levels of no less than 5 g/L.

The allowed IgPro20 maximal infusion rate for all simultaneously used injection sites during the wash-in/wash-out period (Weeks 1–12) was 25 mL/h or lower. A stepwise increase up to 35 mL/h was allowed for the subsequent infusions at the investigator’s discretion, depending on tolerability by the patient.

The main differences between this and the previous two Hizentra® licensing studies [6, 7] were the inclusion of a dedicated IVIG treatment period of 3 months and a pharmacoeconomics analysis.

### Efficacy and Safety Assessments

The primary efficacy endpoint was the geometric mean ratio (GMR) of total serum IgG trough levels on IgPro20 therapy versus that achieved during the mandatory IVIG treatment period. Total serum IgG concentration was measured prior to each IVIG infusion and prior to the IgPro20 infusions at Week 1 and every fourth week thereafter. Comparable trough levels were indicated by a GMR close to 1.

Secondary efficacy endpoints included the annualized rates of SBI (according to the pre-specified US Food and Drug Administration [FDA] criteria [16]) and both serious and non-serious infections during the SCIG efficacy period. Patients were required to keep patient diaries, from which the investigators extracted the information necessary to evaluate the number of days missed from work/school or unable to perform normal daily activities due to infections and duration of hospitalization due to infections. Duration of antibiotics use for infection prophylaxis and treatment was assessed from concomitant medications documented in the Case Report Form.

Safety endpoints were the number, rate, severity, and relatedness of any AEs per infusion and patient, local tolerability of subcutaneous infusions, and changes in vital signs (diastolic and systolic blood pressure, heart rate, and body temperature) before and after infusions at the study site. Local tolerability was assessed by patients between 24 and 72 h after the end of infusion and was analyzed descriptively by calculating frequency distributions of assessment categories (“very good”, “good”, “fair”, or “poor”).

Changes in blood chemistry (albumin, total bilirubin, creatinine, total protein, lactate dehydrogenase, blood urea nitrogen, alanine aminotransferase, aspartate aminotransferase, and alkaline phosphatase), hematology, and urinalysis and changes in viral safety markers for human immunodeficiency virus (HIV-1 and HIV-2), hepatitis C virus (HCV), and hepatitis B virus (HBV), as compared with baseline assessments, were also recorded.

### Statistical Methods

Sample size calculation to assess the GMR of total serum IgG trough levels during IVIG versus SCIG treatment (primary efficacy endpoint) was based upon an assumed residual standard deviation (SD) of 10 %. A 2-sided 90 % confidence interval (CI) was estimated to extend from 0.93 to 1.07 times the GMR if 15 patients were evaluable for the primary analysis. To account for discontinuations, a total of 25 patients were planned for enrollment in the study.

The intention-to-treat (ITT) analysis included data from all patients treated with IgPro20 during the efficacy period. The per-protocol data set (PPS) included all patients who had received at least six doses of IVIG at 3- to 4-week intervals (pre-study and during the IVIG study period) followed by uniform weekly SCIG infusions until at least Week 16 (Fig. 1), with at least one documented total serum IgG trough level in the efficacy period. The safety analysis was based on the “all treated” (AT) data set comprised of all patients who received at least one IgG (IVIG or IgPro20) dose during any study period.

To ensure correct assessment of the GMR, the primary efficacy analysis was based on the PPS, thus excluding patients with dose deviations of >10 %. The GMR (SCIG efficacy period versus mandatory IVIG treatment) with a 2-sided 90 % CI was calculated using mean log-transformed IgG trough levels averaged by patient and treatment period. Additionally, descriptive statistics were calculated for all IgG trough levels by visit and by treatment period. Analyses of secondary efficacy variables were based on the data set used for ITT analysis and the PPS.

Analyses of safety endpoints were based on the AT set. Baseline measurements refer to the sample taken prior to first mandatory IVIG infusion.

## Results

### Patients

**Fig. 2:**
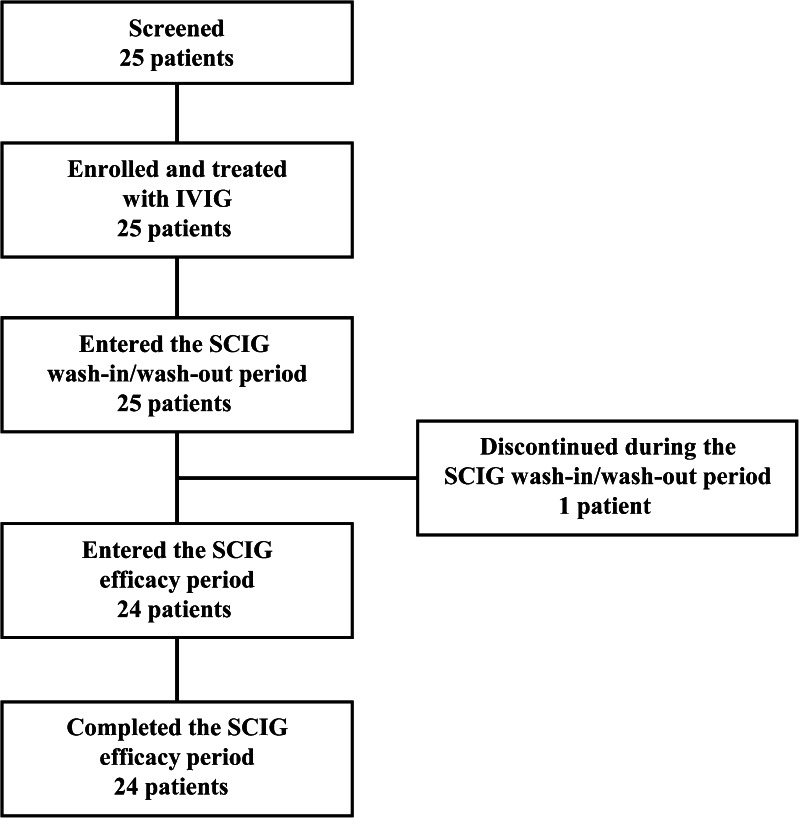
Patient disposition. All 25 screened patients were enrolled into the study and received mandatory IVIG treatment. One patient discontinued from the study during the SCIG wash-in/wash-out period, leaving 24 patients who completed the study

A total of 25 patients were screened and enrolled into the study at nine sites throughout Japan. One patient discontinued from the study during the wash-in/wash-out period due to relocation, leaving 24 patients who completed the study (Fig. 2).

The mean (SD) age of the patients was 20.5 (13.5) years; 11 patients were ≤16 years of age. Fifteen patients (62.5 %) were male (Table I).

**Table I:** Demographic characteristics of patients

Patients	ITT	PPS
Total number of patients	24	21
Gender, n (%)		
Male	15 (62.5)	14 (66.7)
Female	9 (37.5)	7 (33.3)
Age [years], median (range)	17.5 (3–58)	19.0 (3–58)
Age group, n (%)		
<2 years	0	0
≥2 to <12 years	7 (29.2)	4 (19.0)
≥12 to <16 years	4 (16.7)	4 (19.0)
≥16 to <65 years	13 (54.2)	13 (61.9)
≥65 years	0	0
Asian, n (%)	24 (100)	21 (100)
Body weight [kg], median (range)	44.8 (13–105)	48.9 (13–105)
BMI [kg/m^2^], median (range)	18.2 (15–33)	18.2 (15–33)
Type of PID, n (%)		
CVID	10 (41.7)	9 (42.9)
XLA	12 (50.0)	11 (52.4)
ARAG	1 (4.2)	0
VHyper IgM syndrome	1 (4.2)	1 (4.8)

*ARAG* autosomal recessive agammaglobulinemia, *BMI* body mass index, *CVID* common variable immunodeficiency, *ITT* intention-to-treat, *n* number of patients, *PID* primary immunodeficiency, *PPS* per-protocol data set, *XLA* X-linked agammaglobulinemia

The most common PID conditions were X-linked agammaglobulinemia (XLA; 12 patients [50 %], including one well-documented rare female case [17]) and common variable immunodeficiency (CVID; 10 patients [41.7 %]).

### Study Drug Administration

All 25 patients received the intended three IVIG infusions and at least one dose of IgPro20, of which 24 patients received the intended 12 infusions during the SCIG wash-in/wash-out period and another 12 infusions during the efficacy period.

The mean (SD) of individual median weekly IgPro20 doses during the SCIG efficacy period was 87.8 (35.2) mg/kg body weight (bw; Table II). The mean (SD) of the weekly equivalents of individual IVIG doses was 77.3 (30.5) mg/kg bw. An increase from the planned dose by >10 % at any time during the study was documented in 12 patients (48 %).

**Table II:** Weekly IgG doses by treatment period (ITT)

Treatment period	IVIG^a^	SCIG wash-in/wash-out	SCIG efficacy
Total number of infusions	72	288	288
IgG dose, mg/kg bw			
Mean (SD)	77.3 (30.5)	82.2 (33.4)	87.8 (35.2)
Median	73.00	72.98	77.82
Range, min–max	21.5–144.3	26.4–177.8	26.7–172.7

^a^Weekly equivalent dose across both application schedules (every 3 weeks and every 4 weeks) was calculated based on individual infusions

*bw* body weight, *ITT* intention-to-treat, *IVIG* intravenous immunoglobulin, *SCIG* subcutaneous immunoglobulin, *SD* standard deviation

The mean (range) of the individual infusion rates was 22.90 mL/h (13–25 mL/h) during the SCIG wash-in/wash-out period and 25.35 mL/h (12–35 mL/h) during the SCIG efficacy period. The majority of infusions (74.0 %) during the SCIG efficacy period were home-based.

### Efficacy

#### Primary Efficacy Endpoint

**Fig. 3:**
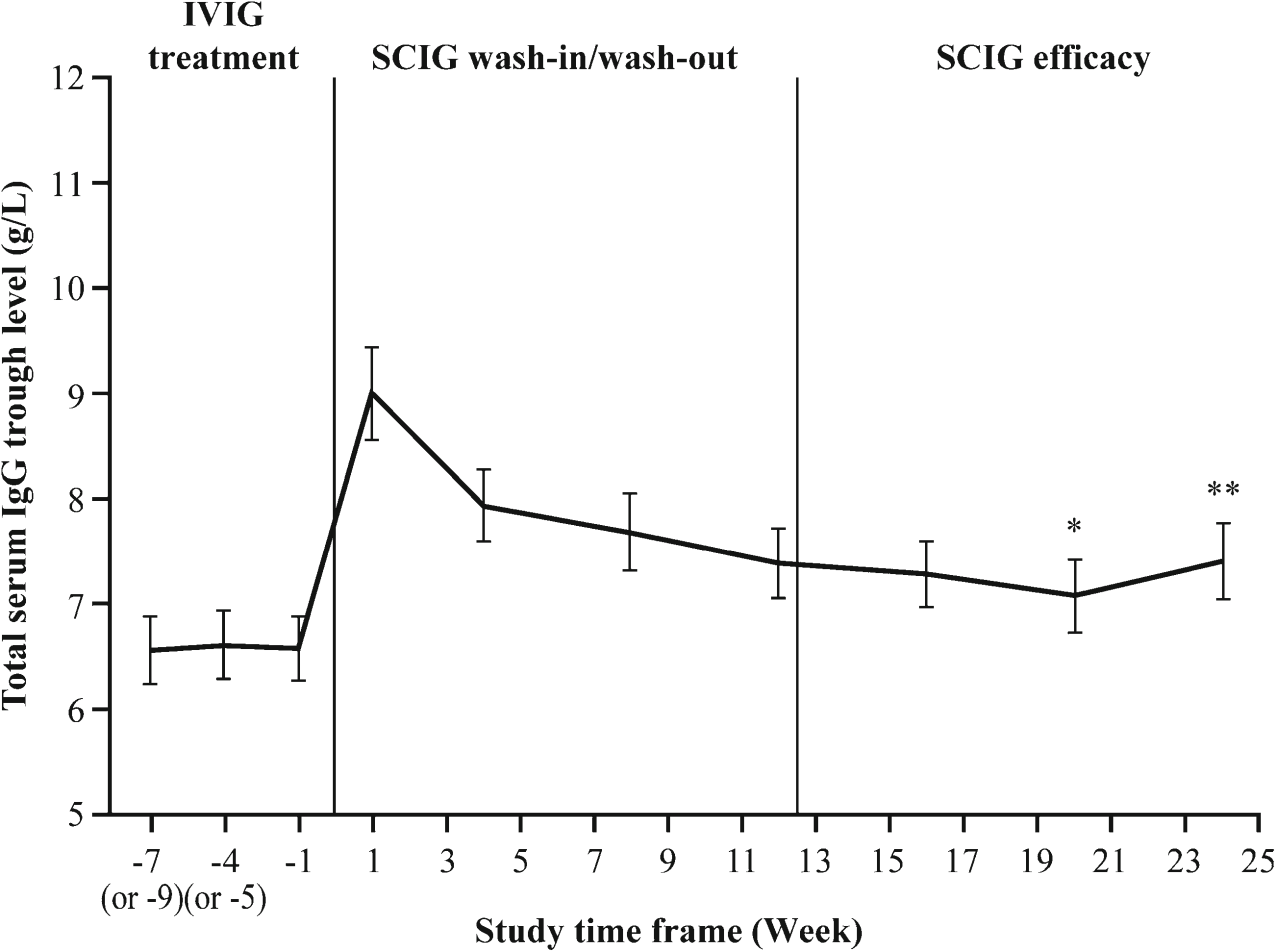
Mean total serum IgG trough levels (PPS). Mean total serum IgG trough levels are shown for the PPS (*N* = 21). The number of patients with available IgG levels differed from the original patient number in the PPS at Week 20 (*N* = 20; ^*^) and Week 24 (*N* = 18; ^**^). *Error bars* represent the standard error of the mean

The primary objective of the study was met, as the GMR of serum IgG trough levels after dose-equivalent switch from IVIG to SCIG was close to 1, with the CI within the accepted equivalence range of 0.80 to 1.25 (1.09 in the PPS [90 % CI: 1.06–1.13]). The mean (SD) IgG trough levels slightly increased from 6.53 (1.40) g/L in the IVIG period to 7.15 (1.51) g/L in the SCIG efficacy period (Fig. 3).

The ITT analysis confirmed these results.

#### Secondary Efficacy Endpoints 

No SBIs were reported at any time during the study.

Rates of infection were maintained at a low level during the SCIG efficacy period, with 11 patients (52.4 %) experiencing an infection (annualized rate of 2.98 infections per patient/year; Table III). Seven patients (33.3 %) missed a total of 19 days from school/work/kindergarten due to infection (3.48 days per patient/year). One patient (4.8 %) was hospitalized during the SCIG efficacy period due to bacterial infection of moderate severity, for a total of 3 days (0.55 days of hospitalization per patient/year); for details see the Safety section.

**Table III:** Summary of secondary efficacy endpoints (PPS)

Secondary efficacy endpoint	Number of patients (%)	Number of events/days (annualized rate)
Total number of patients or exposure days (diary days^a^)	21	1,840 (1,990)
SBIs	0	0
Infection episodes	11 (52.4)	15 (2.98)
Days with antibiotics for infection prophylaxis	5 (23.8)	422 (83.71)
Days with antibiotics for infection treatment	13 (61.9)	458 (90.85)
Days hospitalized due to infections^a^	1 (4.8)	3 (0.55)
Days missed from work/school or unable to perform normal activities due to infections^a^	7 (33.3)	19 (3.48)

^a^As per “diary days” based on analysis of patients’ diaries. *SBI* serious bacterial infection; *PPS* per-protocol data set

A total of 16 patients (76.2 %) were treated with antibiotics on 844 days during the SCIG efficacy period, resulting in an annualized rate of 167.42 days of antibiotic use per patient/year. Antibiotics were used for both treatment of an AE (10 patients [47.6 %]) and for prophylaxis (5 patients [23.8 %]).

### Safety

#### Overall Adverse Events

In the SCIG wash-in/wash-out and SCIG efficacy periods, 24 of the 25 patients (96.0 %) had a total of 269 AEs, with an overall AE rate of 0.461 AEs per infusion. All AEs were of mild or moderate severity, with only 5 AEs (0.009 AEs per infusion) in 3 patients (12.0 %) being of moderate severity. The most common AEs were local reactions (80.0 %), followed by infections, the most common being nasopharyngitis (44.0 %), upper respiratory tract infection (20.0 %), and gastroenteritis (16 %). All other AEs each occurred in less than 4 patients (≤12 %).

During the mandatory IVIG treatment period, 22 patients (88.0 %) experienced 49 AEs (0.653 AEs per IVIG infusion).

#### Temporally Associated and Related Adverse Events

During SCIG treatment, 21 patients (84.0 %) had a total of 175 AEs that were considered by the investigator at least possibly related to the study drug (0.300 AEs per infusion) and 23 patients (92.0 %) experienced 203 AEs that were temporally associated, i.e., occurred within 72 h after an SCIG infusion (0.348 AEs per infusion; Table IV). The majority of the causally-related AEs (91.3 %) and both causally- and temporally-related AEs (91.7 %) were mild local reactions.

**Table IV:** Most common related AEs during the entire SCIG treatment period (SCIG wash-in/wash-out and SCIG efficacy periods) by preferred term (AT)

Preferred term	At least possibly related		At least possibly related and temporally associated events (72 h)	
	Number of patients (%)	Number of events (rate per infusion)	Number of patients (%)	Number of events (rate per infusion)
Total number of patients or infusions	25	584	25	584
Any preferred term	21 (84.0)	175 (0.300)	21 (84.0)	170 (0.291)
Local reactions^a^	20 (80.0)	160 (0.274)	20 (80.0)	156 (0.267)
Skin and subcutaneous tissue disorders	2 (8.0)	9 (0.015)	2 (8.0)	9 (0.015)
Gastrointestinal disorders	1 (4.0)	1 (0.002)	1 (4.0)	1 (0.002)
Investigations	1 (4.0)	1 (0.002)	0 (0.0)	0 (0.000)
Vascular disorders	1 (4.0)	1 (0.002)	1 (4.0)	1 (0.002)

^a^Based on 16 MedDRA preferred terms

*AE* adverse event, *AT* all treated data set, *SCIG* subcutaneous immunoglobulin

In the IVIG treatment period, 1 patient (4.0 %) had at least 2 possibly related AEs (malaise and pyrexia; 0.027 AEs per infusion) and 10 patients (40.0 %) had 12 temporally associated AEs (0.160 AEs per infusion).

None of the infusions was stopped due to an AE. The IgPro20 infusion rate was increased at the investigator’s discretion in 8 patients. None of the AEs reported in these patients could be associated with the increased infusion rate.

#### Serious Adverse Events

During the SCIG treatment, one serious AE (SAE) of moderate severity was reported (0.002 SAEs per SCIG infusion) that was considered by the investigator unrelated to study drug. A 22-year-old male with XLA experienced bacterial infection that was reported as an SAE because of the necessity of hospitalization. The patient was treated with antibacterials and the infection resolved after 15 days. This infection was not considered an SBI, as it did not meet the pre-specified US FDA criteria. No SAEs were reported during the IVIG treatment period.

There were no deaths or AEs resulting in discontinuation of treatment in this study.

#### Local Reactions

Local tolerability of 85.4 % of SCIG infusions was assessed by the patients as “very good” or “good”. In no case was the local tolerability assessed as “poor”.

AEs of local reactions occurred in 20 patients (80.0 %), with an overall rate of 0.277 events per infusion. Almost all local reactions (97.5 %) were temporally associated with SCIG infusion. The rate of local reactions decreased over time from 0.389 events per infusion during the SCIG wash-in/wash-out period to 0.163 events per infusion during the SCIG efficacy period. The overall rate of local reactions related to home-based infusions during the efficacy period was comparable with that related to infusions at investigational site (0.178 events per infusion versus 0.120 events per infusion, respectively).

#### Vital Signs, Laboratory Parameters, and Viral Safety

No consistent or clinically relevant changes in vital signs were reported in this study.

Median values and ranges of hematology, blood chemistry, and urinalysis did not show any relevant changes over time.

Viral safety screening for HIV-1, HIV-2, HCV, and HBV found no positive viral markers at either baseline or 12–17 weeks following the final SCIG infusion.

## Discussion

Primary efficacy analysis of this study demonstrated that IgPro20 administered by the subcutaneous route in uniform weekly doses was an effective treatment in both adult and pediatric Japanese patients with PID. A dose-equivalent switch to SCIG 20 % was effective in maintaining total serum IgG trough levels equal to or above those achieved on the previous IVIG therapy (Fig. 3). The annualized rate of infection during the SCIG efficacy period (2.98 infections/patient/year) was in line with that observed in previous studies in Europe and in the US (2.76–5.18 infections/patient/year), as were the number of days missed from school/work/daycare and days spent in hospital [6, 7, 12]. The mean weekly dose of IgPro20 during the SCIG efficacy period was lower in the Japanese population than that seen in Europe and, in particular, the US [6, 7], probably due to the specifics of local treatment practices including limits on maximum dose allowed per patient.

This is the first prospective study of SCIG to incorporate a mandatory IVIG treatment period into the trial design, allowing for a more stringent comparison of SCIG and IVIG treatments than previous IgPro20 studies. As all patients had received at least 3 IVIG infusions at a stable dose before enrolling in the study, their IgG levels were expected to be at steady state by the end of the mandatory IVIG period. A 12-week SCIG wash-in/wash-out period ensured that the IVIG treatment did not affect serum IgG trough levels during the steady state SCIG efficacy period. SCIG dose adjustments during the wash-in/wash-out period were allowed, but were unlikely to affect the outcome of the study, as the minimal target serum IgG trough level (5.0 g/L) was lower than that achieved during the IVIG treatment period (6.53 g/L).

The male/female imbalance in the patient demographics can be explained by a large share of XLA patients in the study population. Of interest, this subgroup included one well-documented case of XLA in a female patient [17].

Higher rates of antibiotic use in this study compared with the same outcome in SCIG studies conducted outside Japan are likely associated with generally higher administration of antibiotics in Japan [18–20].

IgPro20 was well tolerated. No SAEs related to its administration were reported during the study. One SAE (bacterial infection; etiologic agent not identified) of moderate severity reported during the SCIG efficacy period occurred due to accidental infection and was considered unrelated to the study drug.

The overall incidence of AEs including local reactions during the SCIG wash-in/wash-out and SCIG efficacy periods (0.461 AEs per patient/year affecting 96 % of patients) was comparable with the overall AE rates in other SCIG studies in Europe and in the US (0.288–0.773 AEs per patient/year affecting 98–100 % of patients) [6, 7]. Mild or moderate local reactions (swelling, soreness, redness, and induration) are generally expected when relatively large volumes of IgG are infused by the subcutaneous route. The overall rates of local reactions reported in this study were in line with previous SCIG studies [6, 7, 11, 12].

## Conclusions

Weekly SCIG treatment with Hizentra® was effective in pediatric and adult Japanese patients with PID requiring IgG replacement therapy. The treatment was well tolerated and demonstrated a highly favorable risk-benefit profile.
